# Effects of Psycho-Cardiology Model-Based Psychological and Behavioural Management on Negative Emotions and Sleep Disorders in Patients With Resistant Hypertension Complicated by Anxiety and Depression

**DOI:** 10.62641/aep.2241

**Published:** 2026-10-15

**Authors:** Liyan Zhu, Weihua Hu

**Affiliations:** ^1^Department of Cardiology, Tonglu Hospital, Hangzhou First People's Hospital Tonglu Campus, 311500 Hangzhou, Zhejiang, China

**Keywords:** behavioral medicine, resistant hypertension, anxiety, depression, sleep disorders, psychosomatic medicine, psycho-cardiology model

## Abstract

**Background:**

To explore the effect of psycho-cardiology model-based psychological and behavioural management on top of routine cardiovascular treatment on negative emotions and sleep disorders in patients with resistant hypertension complicated by anxiety and depression.

**Methods:**

This retrospective study reviewed electronic medical records and follow-up data of patients treated in the Department of Cardiology of Tonglu Hospital, Hangzhou First People's Hospital Tonglu Campus between August 2022 and May 2025. A total of 126 patients diagnosed with resistant hypertension complicated with anxiety and depression who met the predefined inclusion criteria were enrolled. Based on whether the psycho-cardiology model-related assessment and psychological and behavioural management were systematically documented in their medical records, the patients were divided into a psycho-cardiology group (n = 63) and a regular group (n = 63). Changes in anxiety, depression, sleep quality, and blood pressure level were compared between the two groups during the follow-up period.

**Results:**

At the end of follow-up, the Hamilton Anxiety Rating Scale, Hamilton Depression Rating Scale, and Pittsburgh Sleep Quality Index scores of the psycho-cardiology group were significantly lower than those of the regular group (*p* < 0.05); Reductions in systolic and diastolic blood pressure were greater in the psycho-cardiology group than in the regular group (*p* < 0.05).

**Conclusions:**

In the long-term management of patients with resistant hypertension and anxiety and depression, the psycho-cardiology model combined with psychological and behavioural management was associated with milder negative emotions, better sleep status, improved autonomic function and more stable blood pressure profiles in this retrospective cohort.

## Introduction

Resistant hypertension refers to a subtype of hypertension that is difficult to manage. It is generally defined as the state in which blood pressure still fails to reach the target level when ≥3 antihypertensive drugs with different mechanisms of action are prescribed at appropriate doses. These patients are exhibit clinical characteristics such as large fluctuations in blood pressure, circadian rhythm disorders and a significantly increased risk of cardiovascular events, which have an adverse effect on long-term prognosis [1, 2, 3].

With the increasing recognition of the biopsychosocial model, the role of psychological and emotional factors in the occurrence, development and control of hypertension has gradually attracted attention. Research indicates that approximately one-third of people with hypertension experience varying degrees of anxiety and depression, and the prevalence of anxiety and depression among those with hypertension is five times higher than in people with normal blood pressure [4]. Anxiety and depression can contribute to the pathophysiological process of hypertension through multiple pathways, including activating the sympathetic nervous system, disrupting the function of the hypothalamus-pituitary-adrenal axis, aggravating inflammatory responses and endothelial dysfunction, thereby forming a vicious cycle of emotional imbalance, elevated blood pressure and further deterioration of mood [5].

Sleep disorders are another common and easily overlooked clinical manifestation in patients with hypertension and psychological disorders [6]. Studies have found that poor sleep quality can lead to high blood pressure, a weakened immune system, and mental health issues; depression and anxiety can also cause sleep disorders. Long-term sleep disturbances are closely associated with increased blood pressure variability in hypertensive patients, which may increase the risk of adverse cardiovascular events. This association is particularly significant in patients with poorly controlled hypertension [7, 8].

The psycho-cardiology model emphasizes the bidirectional relationship between cardiovascular disease and psychological disorders, and advocates that the psychological state, behaviour patterns and lifestyle characteristics be assessed and managed in the process of cardiovascular disease management to improve both disease control and mental health [9]. In this model, psychological and behavioural assessments are regarded as important components of cardiovascular risk assessment [10].

Therefore, this study retrospectively analysed the clinical data of patients with resistant hypertension and anxiety and depression treated at our hospital from August 2022 to May 2025 to compare changes in negative emotions, sleep quality and blood pressure levels under different management models. The findings may provide clinical basis for the comprehensive assessment and long-term management of these patients.

## Materials and Methods

### General Information

This retrospective study reviewed electronic medical records and follow-up data of patients treated in the Department of Cardiology of Tonglu Hospital, Hangzhou First People’s Hospital Tonglu Campus between August 2022 and May 2025. A total of 126 patients diagnosed with resistant hypertension and comorbid anxiety and depression who met the predefined inclusion criteria were enrolled. This study was approved by the Medical Ethics Committee of Tonglu Hospital, Hangzhou First People’s Hospital Tonglu Campus (approval No. KJ-2026-14) and was conducted in accordance with ethical guidelines for retrospective observational research. The requirement for informed consent was waived by the ethics committee due to the retrospective use of de‑identified medical records. The research followed the guiding principles of the Declaration of Helsinki.

Patients were divided into a psycho-cardiology group (n = 63) and a regular group (n = 63) based on whether their medical records documented psycho-cardiology-related assessments and psychological and behavioural management. Group assignment was determined by the joint diagnosis and treatment team of cardiology and psychiatry based on the predefined psycho-cardiology documentation criteria. If the medical record systematically documented the core elements of psychological scale assessment, concurrent monitoring of mood and blood pressure, sleep and behavioural intervention guidance related to the psycho-cardiology model, the patient was assigned to the psycho-cardiology group; those without systematic records of the above elements were included in the regular group. All included patients completed initial assessments and treatment plans during hospitalization and were subsequently placed under outpatient follow-up management after discharge.

### Selection Criteria

#### Inclusion Criteria

(1) Met the diagnostic criteria for resistant hypertension in the Chinese Guidelines for the Prevention and Treatment of Hypertension (latest edition) [11]; (2) Diagnosed by a psychiatrist with anxiety disorder and depression disorder, with the scale assessment met the following criteria: Hamilton Anxiety Rating Scale (HAMA) score ≥14 points (mild and moderate anxiety, 14–28 points) [12], and Hamilton Depression Rating Scale-17 (HAMD-17) score≥8 points (mild and moderate depression, 8–24 points) [13]. (3) Age ≥18 years; (4) Complete medical records during the treatment and follow-up period; (5) Follow-up duration ≥6 months, with complete follow-up records.

#### Exclusion Criteria

(1) Patients with secondary hypertension (e.g., renal, endocrine or drug-induced hypertension); (2) Patients with severe mental disorders or significant cognitive impairment who are unable to complete standardized psychological assessments; (3) Patients with severe physical illnesses, malignant tumours or a life expectancy of less than 1 year; (4) Those with a confirmed diagnosis of sleep apnoea syndrome without standardized assessments or without complete records.

### Methods

The researchers uniformly extracted clinical data for statistical analysis. The evaluators of the research results were all third-party researchers who had received professional training. To minimize potential bias in the retrospective analysis, these researchers were blinded to patient grouping during data extraction and outcome evaluation, because the grouping information was not disclosed in the extracted datasets.

#### Pharmacological Treatment

Both groups received standardized antihypertensive pharmacological treatment in accordance with guideline recommendations. The drug regimen consisted of triple combination therapy: a calcium channel blocker (amlodipine 5–10 mg once daily), a renin‑angiotensin system blocker (valsartan 80–160 mg once daily), and a thiazide-like diuretic (indapamide 1.5 mg once daily). If blood pressure remained uncontrolled, spironolactone (25–50 mg once daily) was added as fourth‑line therapy. All adjustments were individualized based on heart rate, renal function, and comorbidities [11, 14]. Twelve patients in the psycho-cardiology group and 10 patients in the regular group had minor dosage adjustments of antihypertensive drugs due to blood pressure control conditions. No changes in the types of drugs were documented. The subsequent statistical analysis of blood pressure changes has included the adjustments in antihypertensive treatment as confounding factors in the model for correction.

#### Regular Group

Patients in the regular group received routine cardiovascular management with blood pressure control as the core. (1) Blood pressure monitoring and basic assessment: all patients received routine blood pressure monitoring during hospitalization. The medical records show that patients’ blood pressure was regularly measured, and the results were recorded. (2) Pharmacological treatment and compliance reminders: Antihypertensive drugs were administered. Patients received medication education regarding antihypertensive drugs and reminded them to take their medication on time and not to miss doses or adjust the dosage on their own. (3) General health education: Patients received routine health education, including low-salt diet recommendations, the principle of appropriate exercise, and smoking cessation and alcohol limitation. The education was mainly conducted verbally. (4) Routine condition observation: Patients were assessed for hypertension-related such as dizziness, headache, and palpitations [15].

#### Psycho-Cardiology Group

The psycho-cardiology group simultaneously received cardiovascular treatment and psychological and behavioural management. All patients in this group had received a set of measures that were consistent with the “Standardized Implementation Plan for Psycho-Cardiology Interventions” as part of routine clinical care [10].

(1) Multidisciplinary team and disease education: A multidisciplinary team including cardiologists, rehabilitation physicians, nutritionists, psychologists, general practitioners, and nurses was established to provide comprehensive care. Patients received structured disease education regarding the definition, grading, treatment methods, possible complications, and high-risk triggers of their condition, which helped correct misconceptions and improve treatment adherence. (2) Psychological assessment and intervention: Patients underwent psychological evaluation using the HAMA and HAMD at baseline and during follow-up. Individualized psychological interventions, including support, encouragement, comfort, and counselling, were provided weekly by specialized nurses and psychologists through both in-person and online sessions, with each session lasting approximately 20 minutes. For patients with severe negative emotions, targeted face to face interviews were conducted to identify underlying causes and implement personalized management. (3) Relaxation training and family support: Relaxation techniques such as mindful walking, mindful breathing, mindful meditation, pursed-lip breathing, abdominal breathing, and music relaxation training were delivered. Family members were encouraged to communicate more with patients, provide emotional support, and participate in care. Caregivers received education on the positive impact of family support on recovery and were guided to provide appropriate assistance, thereby helping patients build treatment confidence. (4) Personalized health records and internet based health education: Personal files were established for each patient, incorporating baseline survey results of the 36-Item Short Form Survey (SF-36) scale, HAMA, HAMD, and relevant clinical data. After discharge, personalized health education content was pushed through an internet platform, covering medication, diet, exercise, lifestyle, and psychological counselling. Patients received regular reminders for outpatient return visits via telephone or internet at 1 week, 1 month, 3 months, and 6 months after discharge. The 6 month follow-up endpoint included reassessment of psychological status, self management ability, medication adherence, cardiac function grading, and occurrence of cardiovascular adverse events. (5) Follow up methods and frequency: All patients were managed using an outpatient follow up method after discharge. Follow-up was conducted via telephone calls and internet based platforms (e.g., WeChat), as documented in the psycho-cardiology model study. The duration of each telephone or online follow-up was typically 10–15 minutes, whereas outpatient follow-up records indicated a full set of examinations including scale assessment and blood pressure re measurement, with a duration of approximately 30 minutes. All the included patients in this study completed the 6-month follow-up throughout the process, with no loss to follow-up and a follow-up integrity rate of 100% [10].

### Observation Indicators

#### Negative Emotional State

Baseline data recorded during hospitalization and the 6-month follow-up data were statistically analysed. The HAMA [12] consists of 14 items, each scored from 0 to 4, yielding a total score of 0 to 56. Higher scores indicate more severe anxiety. The HAMD-17 [13] consists of 17 items, for a total score of 0 to 52. Higher scores indicate more severe depression.

#### Sleep Quality

Baseline data recorded during hospitalization and assessment data at 6-month follow-up were collected. Sleep quality was assessed using the Pittsburgh Sleep Quality Index (PSQI) [16]. The PSQI has a total score ranging from 0 to 21, with higher scores indicating poorer sleep quality.

#### Blood Pressure Level

Systolic and diastolic blood pressure levels at baseline and 6-month follow-up were collected from patient medical records. Standard sitting blood pressure was used, and the average of two consecutive measurements taken between 7:00 and 9:00 AM was used as the analysis data.

#### Medication Adherence

Baseline data recorded during hospitalization and assessment data at 6-month follow-up were statistically analysed. The Morisky Medication Adherence Scale (MMAS-8) [17] was used, with a total score of 0 to 8. A higher score indicates better medication adherence.

#### Quality of Life

The SF-36 scale scores recorded in the medical records were statistically analysed [18]. The assessment dimensions include physical functioning, role physical, bodily pain, general health, vitality, social functioning, role emotional and mental health. The scores for each dimension ranged from 0 to 100. The higher the score, the better the quality of life.

### Statistics

Data analysis was conducted using SPSS 26.0 statistical software (IBM Corp., Armonk, NY, USA) and R 4.3.0 software (R Foundation for Statistical Computing, Vienna, Austria; lme4 package). Firstly, the Shapiro–Wilk test was used to test the normality of continuous data. Continuous data that conformed to a normal distribution were expressed as mean ± standard deviation ($\bar{x}$ ± s), whereas continuous data that did not conform to a normal distribution were expressed as median [interquartile range] (M [Q1, Q3]), and categorical data were expressed as number (percentage) [n (%)]. For repeated measurements of continuous data at baseline and follow-up, analysis of covariance (ANCOVA) was used to correct for baseline indicators and then compare between groups, and a linear mixed-effects model (LMM) was used to analyse the time-group interaction effect of repeated measurements to verify the robustness of the results; independent sample *t*-tests were used for comparisons between groups for continuous data that conformed to a normal distribution, and paired t-tests were used for comparisons within groups before and after. Mann-Whitney U tests were used for comparisons between groups for continuous data that did not conform to a normal distribution, and χ^2^ tests were used for categorical data. A *p* value <0.05 was considered statistically significant. For continuous variables in the independent samples t-test between groups, the effect size was expressed by Cohen’s d, calculated using the formula as follows: $d=\frac{{\bar{x}}_{1}-{\bar{x}}_{2}}{S_{\text{pooled}}}$, where $S_{\text{pooled}}=\sqrt{\frac{\left(n_{1}-1\right)\,S_{1}^{2}+\left(n_{2}-1\right)\,S_{2}^{2}}{n_{1}+n_{2}-2}}$, standing for the combined standard deviation. The 95% confidence interval was estimated using the Bootstrap method (with 1000 repeated samplings).

## Results

### Comparison of Baseline General Information Between the Two Groups

There were no significant differences between the psycho-cardiology group and the regular group in terms of gender, age, duration of hypertension, body mass index, and comorbidities (*p* > 0.05), indicating that baseline data are comparable between groups, see Table 1 for details.

**Table 1.** **Comparison of general data between the two groups**.

Index		Psycho-cardiology group (n = 63)	Regular group (n = 63)	t/χ^2^	*p*
Gender [n (%)]				0.127	0.722
	Male	33 (52.38)	31 (49.21)		
	Female	30 (47.62)	32 (50.79)		
Age (years, $\bar{x}$ ± s)		60.81 ± 8.43	61.91 ± 9.05	–0.704	0.483
Duration of hypertension (years, $\bar{x}$ ± s)		12.94 ± 5.11	12.32 ± 4.71	0.708	0.480
BMI (kg/m^2^, $\bar{x}$ ± s)		25.83 ± 1.15	25.55 ± 1.31	1.275	0.204
Comorbid type 2 diabetes [n (%)]		18 (28.57)	19 (30.16)	0.038	0.845
Comorbid coronary artery disease [n (%)]		17 (26.98)	16 (25.40)	0.041	0.839
Comorbid dyslipidemia [n (%)]		18 (28.57)	17 (26.98)	0.040	0.842
Mild chronic renal dysfunction [n (%)]		10 (15.87)	11 (17.46)	0.057	0.811

BMI, body mass index.

### Comparison of Negative Emotional States Before and After Treatment in the Two Groups

After treatment, the HAMA and HAMD scores in both groups decreased significantly compared with those before treatment (*p* < 0.05 for all within-group comparisons). However, the scores of the psycho-cardiology group were statistically lower than those of the regular group after treatment (*p* < 0.05), see Table 2 for details. After adjusting for baseline HAMA and HAMD scores using ANCOVA, the scores of the psycho-cardiology group after treatment were significantly lower than those of the regular group (*p* < 0.05). The effect sizes and 95% confidence intervals (CIs) were as follows: HAMA (Cohen’s d = 1.20, 95% CI: 0.81–1.60), HAMD (Cohen’s d = 0.54, 95% CI: 0.17–0.91). After Bonferroni correction (corrected α = 0.0125), the above differences remained statistically significant (*p* < 0.001 for both).

**Table 2.** **Comparison of negative emotional state scores before and after treatment in the two groups (points)**.

Group	n	HAMA		HAMD	
		Before treatment	After treatment	Before treatment	After treatment
Psycho-cardiology group	63	18.72 ± 3.64	11.62 ± 2.48*	16.34 ± 4.12	12.94 ± 3.02*
Regular group	63	18.91 ± 3.58	14.87 ± 2.91*	16.58 ± 4.26	14.68 ± 3.45*
t		0.295	6.747	0.321	3.012
*p*		0.768	<0.001	0.748	0.003

Note: *p < 0.05 compared with pre-treatment levels in this group. HAMA, Hamilton Anxiety Rating Scale; HAMD, Hamilton Depression Rating Scale.

### Comparison of Sleep Quality Before and After Treatment Between the Two Groups

After treatment, the PSQI scores of both groups decreased significantly compared with those before treatment (*p* < 0.05 for all within-group comparisons). However, the PSQI score of the psycho-cardiology group was significantly lower than that of the regular group after treatment (*p* < 0.001), see Table 3 for details. After adjusting for baseline scores using ANCOVA, the PSQI score of the psycho-cardiology group was significantly lower than that of the regular group (*p* < 0.05), with a Cohen’s d of 1.20 (95% CI: 0.81–1.60).

**Table 3.** **Comparison of PSQI scores before and after treatment in the two groups (points)**.

Group	n	Before treatment	After treatment
Psycho-cardiology group	63	12.86 ± 2.71	7.86 ± 1.94*
Regular group	63	12.94 ± 2.63	10.42 ± 2.31*
t		0.168	6.736
*p*		0.867	<0.001

Note: *p < 0.05 compared with pre-treatment levels in this group. PSQI, Pittsburgh Sleep Quality Index.

### Comparison of Blood Pressure Levels Before and After Treatment in the Two Groups

After treatment, both systolic and diastolic blood pressure decreased significantly compared with before treatment (*p* < 0.05 for all within-group comparisons); However, the systolic and diastolic blood pressure levels in the psycho-cardiology group were significantly lower than those in the regular group (*p* < 0.001), see Table 4 for details. After adjusting for baseline blood pressure levels using ANCOVA, the systolic and diastolic blood pressure levels in the psycho-cardiology group were significantly lower than those in the regular group (*p* < 0.05). The effect size Cohen’s d for systolic blood pressure was 1.47 (95% CI:1.06–1.87), and for diastolic blood pressure, Cohen’s d was 1.12 (95% CI:0.73–1.50).

**Table 4.** **Comparison of blood pressure levels before and after treatment in the two groups of patients**.

Group	n	Systolic blood pressure		Diastolic blood pressure	
		Before treatment	After treatment	Before treatment	After treatment
Psycho-cardiology group	63	156.48 ± 12.76	132.48 ± 8.76*	94.62 ± 3.91	82.36 ± 6.41*
Regular group	63	157.82 ± 13.04	145.92 ± 9.54*	95.14 ± 4.18	89.84 ± 6.88*
t		0.583	8.236	0.721	6.314
*p*		0.561	<0.001	0.472	<0.001

Note: *p < 0.05 compared with pre-treatment levels in this group.

### Comparison of Medication Adherence Between the Two Groups Before and After Treatment

After treatment, the MMAS-8 scores of both groups were significantly improved compared with those before treatment (*p* < 0.05 for all within-group comparisons), and the MMAS-8 score of the psycho-cardiology group was significantly higher than that of the regular group (*p* < 0.001), see Table 5 for details. After adjusting for baseline scores using ANCOVA, the MMAS-8 score of the psycho-cardiology group was significantly higher than that of the regular group (*p* < 0.001), with a Cohen’s d of 1.07(95% CI: 0.69–1.44).

**Table 5.** **Comparison of MMAS-8 scores before and after treatment in the two groups (points)**.

Group	n	Before treatment	After treatment
Psycho-cardiology group	63	4.19 ± 0.98	6.76 ± 0.82*
Regular group	63	4.25 ± 1.08	5.73 ± 1.10*
t		–0.346	5.991
*p*		0.730	<0.001

Note: *p < 0.05 compared with pre-treatment levels in this group. MMAS-8, Morisky Medication Adherence Scale.

### Comparison of Quality of Life

The scores of each dimension of SF-36 in the psycho-cardiology group after treatment were significantly higher than those in the regular group (*p* < 0.05). Across all dimensions, between-group effect sizes reflected large to very large intergroup differences, with Cohen’s d ranging from 1.61 to 2.72. Detailed Cohen’s d values and corresponding 95% CIs for each SF-36 subscale are presented in Table 6.

**Table 6.** **Comparison of quality of life (points)**.

Group name	Number of examples	Time	Physical functioning	Role physical	Bodily pain	General health	Vitality	Social functioning	Role emotional	Mental health
Psycho-cardiology group	63	Before treatment	65.42 ± 6.11	66.08 ± 5.94	67.35 ± 5.62	64.91 ± 5.87	66.73 ± 5.58	67.12 ± 5.76	65.84 ± 5.69	66.18 ± 5.52
Regular group	63	Before treatment	64.88 ± 6.27	65.71 ± 6.03	66.94 ± 5.71	64.35 ± 5.79	66.25 ± 5.64	66.78 ± 5.81	65.46 ± 5.73	65.92 ± 5.61
t	–	–	0.490	0.347	0.406	0.539	0.480	0.330	0.373	0.262
*p*	–	–	0.625	0.729	0.685	0.591	0.632	0.742	0.709	0.794
Psycho-cardiology group	63	After treatment	85.13 ± 5.44*	85.12 ± 5.04*	86.86 ± 4.41*	80.07 ± 5.45*	87.27 ± 5.06*	87.42 ± 5.31*	83.82 ± 5.36*	82.96 ± 4.51*
Regular group	63	After treatment	76.05 ± 5.38*	76.88 ± 4.11*	74.83 ± 4.42*	72.11 ± 4.41*	75.15 ± 5.17*	75.07 ± 5.23*	74.17 ± 5.25*	73.96 ± 4.62*
t	–	–	9.420	10.057	15.293	9.012	13.298	13.152	10.209	11.064
*p*	–	–	<0.001	<0.001	<0.001	<0.001	<0.001	<0.001	<0.001	<0.001
Post‑treatment Cohen’s d	–	–	1.68	1.79	2.72	1.61	2.37	2.34	1.82	1.97
95% CI	–	–	1.25–2.10	1.35–2.22	2.23–3.20	1.19–2.02	1.91–2.82	1.88–2.79	1.38–2.25	1.52–2.41

Note: *p < 0.05 compared with pre-treatment levels in this group.

## Discussion

Patients with hypertension are prone to anxiety, depression and other negative emotions due to long-term illness and blood pressure fluctuations, which in turn induce further blood pressure instability, creating a vicious cycle [19]. Negative emotions can also adversely affect the cardiovascular system through multiple neuroendocrine pathways, further aggravating the difficulty of blood pressure control and making the disease present obvious psychological and physical characteristics. From a pathophysiological perspective, anxiety and depression can lead to continuous activation of the sympathetic nervous system, decreased vagal tone, increased heart rate, increased peripheral vascular resistance, and disruption of the diurnal rhythm of blood pressure [20]. This is supported by evidence of increased sympathetic nerve activity and reduced baroreflex sensitivity observed in hypertensive patients during emotional stress (e.g., the “white-coat” effect). This sympathetic overdrive can further impair blood pressure regulation and disrupt sleep structure. Therefore, resistant hypertension combined with anxiety and depression is not merely a coexistence of conditions, but rather a clear bidirectional association [21].

The present study showed that the HAMA and HAMD scores in the psycho-cardiology group decreased significantly after treatment compared with those in the regular group (*p* < 0.05). This may be because the psycho-cardiology process not only provided timely attention to patients with obvious emotional problems but also incorporated psychological assessment into the routine process. Through scaled assessment and continuous documentation, the anxiety and depression status were continuously monitored [22]. This process of systematic assessment and documentation helps patients gradually face their emotional problems and reduce the continuous psychological burden caused by emotional suppression or neglect. In addition, providing patients with a comfortable environment and necessary disease related education, along with psychological support, can help alleviate negative emotions such as fear. This comprehensive care helps patients better cope with their condition and treatment, thereby improving overall disease management [23].

In addition, the PSQI score in the psycho-cardiology group was significantly lower than that of the regular group after treatment (*p* < 0.05). Since anxiety and depression are important contributors to difficulty falling asleep, nighttime awakening and early awakening, in turn, aggravate mood fluctuations, forming a vicious cycle. In the management of mental stress-induced hypertension, improving sleep quality is recognized as an important component of non‑pharmacological interventions. Behavioural strategies such as sleep hygiene education, regulation of work‑rest schedules, and modification of pre-sleep habits can help patients establish a more stable sleep rhythm. Better sleep continuity and efficiency may in turn alleviate mood disturbances caused by insufficient sleep, thereby contributing to blood pressure control. These findings are consistent with the psycho-cardiology model practiced in China, which emphasizes the bidirectional relationship between hypertension and psychological disorders [24].

The current study also showed that the systolic and diastolic blood pressure levels in the psycho-cardiology group were lower than those in the regular group after treatment (*p* < 0.05). This is consistent with findings showing that lower trait anxiety (reflecting greater emotional stability) is associated with lower resting muscle sympathetic nerve activity and lower blood pressure [25]. Similarly, sleep disturbances have been associated with increased blood pressure variability and impaired nocturnal blood pressure dipping, suggesting that better sleep quality may be related to more favourable diurnal blood pressure rhythms [8]. During the follow-up process, staff recorded blood pressure changes concurrently with patients’ psychological status and sleep status, which may have helped patients recognize the association between psychological state and blood pressure, thereby reducing excessive tension caused by short-term fluctuations in blood pressure. In this virtuous cycle, stable blood pressure control, in turn, enhances patients’ confidence in disease management and further consolidates the improvement of psychological status and sleep quality [26].

The medication adherence results showed that the MMAS-8 score in the psycho-cardiology group was significantly higher than that in the regular group (*p* < 0.05). The study showed that patients’ perception of hypertension—particularly their beliefs about the consequences of the disease, personal control over the condition, and recognition of symptoms—was positively associated with medication adherence. These findings suggest that when patients have a stronger and more accurate understanding of their illness, they are more likely to maintain more active medication behaviour, which may contribute to better overall adherence [27].

Further research results showed that the SF-36 scale scores of the psycho-cardiology group were higher than those of the regular group (*p* < 0.05). This may be because improved psychological status (reduced anxiety and depression), better sleep quality, and stable blood pressure control can work together to enhance patients’ physical function and social interaction [28].

Studies suggest that for patients with poorly controlled hypertension and comorbid anxiety, depression, or sleep disorders, should undergo routine screening of psychological status and sleep quality, rather than wait until they report significant psychological discomfort. Routinely using standardized scales to assess anxiety, depression, and sleep status during daily diagnosis and follow-up helps identify high-risk patients early, providing a basis for subsequent treatment. Concurrent documentation of psychological status, sleep patterns, and blood pressure changes helps healthcare professionals gain a more comprehensive understanding of the psychological and behavioural factors behind blood pressure fluctuations, thus avoiding treatment strategies that rely solely on increasing medication or frequently changing treatment plans.

Furthermore, the psychological and behavioural treatment measures used in this study are relatively simple, primarily delivered through follow-up, and do not require invasive procedures or additional medication burden, making it feasible for broader clinical application. In clinical practice, the existing cardiology treatment and follow-up system can be leveraged to integrate the concept of “psycho-cardiology” into routine management processes through standardized questioning, guidance on emotional expression, sleep behaviour instruction, and lifestyle modification. This allows for the synergistic advancement of pharmacological treatment, psychotherapy, and behavioural management. This management model was correlated with better mood, sleep and sustained blood pressure outcomes, accompanied by higher medication adherence and self-management levels in this retrospective analysis. Therefore, the results of this study provide new insights into clinical treatment strategies for patients with resistant hypertension. While adhering to guideline-recommended pharmacological treatment, psychological and behavioural management should be incorporated into routine care as an important auxiliary management tool into routine practice, shifting from a single blood pressure control goal to a holistic psycho-cardiology management model to achieve more comprehensive and stable treatment outcomes.

This study has several limitations. It is a retrospective observational study. Patients were non-randomly grouped based on whether the medical records contained the “psycho-cardiology” components and whether the psycho-cardiology assessment and psychological and behaviour management were systematically recorded. This grouping method is prone to selection bias, information bias, and residual confounding. Most critically, group allocation depended on the completeness and systematic nature of documentation in routine medical records rather than on an independent clinical assignment process. Consequently, the observed differences between groups may partly reflect variations in documentation quality (e.g., more detailed recording in one group) rather than true differences in clinical management. Although baseline characteristics were comparable, unmeasured confounding factors and potential recording bias cannot be excluded. Moreover, this study design does not permit causal inference and can only demonstrate the associations between the indicators. The effect sizes observed in this study were relatively large (Cohen’s d ranging from 0.54 to 2.72), which may suggest clinical relevance; however, they should be interpreted with caution. Several factors inherent to the retrospective design may have contributed to these inflated estimates: (1) selection bias due to non-randomized grouping; (2) information bias, because group assignment depended on documentation completeness, meaning that patients with more detailed records (who may have received more attentive care) were assigned to the psycho-cardiology group; and (3) potential confounding by unmeasured variables such as socioeconomic status, health literacy, and baseline psychological resilience. Therefore, the observed effect sizes may overestimate the true clinical benefits of the psycho-cardiology model and should not be directly compared with those reported in prospective randomized trials. The wide 95% confidence intervals may also reflect the limited sample size and sampling variability.

## Conclusions

The psycho-cardiology model, by simultaneously addressing psychological, sleep, and behavioural factors, may help interrupt the vicious cycle of psychological imbalance, sleep disorders, and blood pressure fluctuations in patients with resistant hypertension. In this retrospective observational study, psycho-cardiology-based management was associated with improvements in anxiety, depression, sleep quality, blood pressure control, medication adherence and quality of life. These findings suggest potential benefits for the long-term management of the disease; however, because of the retrospective observational design, causal inferences cannot be drawn, and the results should be interpreted as correlational. Prospective studies are needed to confirm these associations.

## Availability of Data and Materials

The datasets used and/or analysed during the current study were available from the corresponding author on reasonable request.
